# Facile Synthesis of Silver Nanoparticles with High Antibacterial Activity

**DOI:** 10.3390/ma11122498

**Published:** 2018-12-08

**Authors:** Anni Feng, Jiankang Cao, Junying Wei, Feng Chang, Yang Yang, Zongyuan Xiao

**Affiliations:** Department of Chemical and Biochemical Engineering, College of Chemistry and Chemical Engineering, Pen-Tung Sah Institute of Micro-Nano Science and Technology, Xiamen University, Xiamen 361005, China; 5anne5@sina.com (A.F.); 20620161151564@stu.xmu.edu.cn (J.C.); grace_junying@163.com (J.W.); changwei345@icloud.com (F.C.)

**Keywords:** microemulsion, silver nanoparticle, silver nanowire, antibacterial activity

## Abstract

We report on a reverse microemulsion method for the synthesis of silver nanocrystals and examine their antibacterial activities. As the molar ratio of water to sodium bis(2-ethylhexyl) sulfosuccinate (AOT) increases to 25, a morphology transition from a sphere-like nanocrystal to a wire-like one was observed. For both the gram-negative and gram-positive bacteria, the wire-like silver nanocrystal showed higher antibacterial activities. We conclude that the morphology of silver nanocrystals dominates their antibacterial activity.

## 1. Introduction

The design, synthesis, and study of new antibacterial materials are of great significance in both materials’ chemistry and public health. Since the 1990s, metal nanocrystals have emerged as promising materials as they have vast applications in catalysis [1,2,3,4,5,6], trace detection [7,8,9,10,11], electronics [12,13,14,15,16], and biomaterials [17,18,19,20,21,22]. Among the metal nanocrystals, silver nanocrystals have attracted much interest in recent years due to their antibacterial activities [23,24,25,26,27,28,29,30,31,32], and they have been widely used in various consumer products such as textiles, food storage containers, laundry additives, paints, and food supplements [33,34,35,36,37,38,39,40,41,42]. A large number of methods for fabricating silver nanocrystals have been developed, including chemical reduction [43,44,45,46,47,48], electrochemical reduction [49,50,51,52,53], template method [54,55], photocatalytic reduction [56,57,58], break junction [59], and biochemical reduction [60,61,62,63,64,65].

In the present work, a simple, economic, and effective approach has been developed to prepare silver nanocrystals on the basis of the reverse microemulsion method. In a microemulsion solution containing n-heptane, sodium bis(2-ethylhexyl) sulfosuccinate (AOT), and silver nitrate, silver cations were reduced to silver nanocrystals through the addition of an ascorbic acid solution. The as-prepared silver nanocrystals were characterized thoroughly by scanning electron microscopy (SEM), dynamic light scattering (DLS), and ultraviolet-visible spectroscopy (UV-vis), where a morphological transition from sphere to wire was observed. Their disinfection efficiency was investigated in detail via measurement of the minimum inhibitory concentration (MIC) and the minimum bactericidal concentration (MBC).

## 2. Experimental Details

### 2.1. Reagents

AOT (>96%), ascorbic acid (>99.7%), and silver nitrate (>99.8%) were purchased from China Medicine (Group) Shanghai Chemical Reagent Corp. (Shanghai, China). AOT was purified according to the previous report [66]. All the other reagents and chemicals were of analytical grade and used as received.

### 2.2. Characterization of Silver Nanocrystals

UV-Vis spectra were recorded with a UV-2010 UV-vis spectrophotometer (Hitachi, Tokyo, Japan) to find the optimized mass fractions of AOT (W_AOT_), and to confirm the formation of silver nanocrystals. Scanning electron microscope (SEM) images were collected by a Hitachi S-4800 electron microscope (Tokyo, Japan) after the samples were dropped on to silicon wafers. The powdered samples were subjected to X-ray diffraction (XRD) studies on an X’pert PRO XRD (Malvern Panalytical, Malvern, UK) to identify their structures. The diameter of the synthesized silver nanocrystals was probed by dynamic light scattering (DLS) with a Brookhaven 90 Plus (Brookhaven Instruments Corp., New York, NY, USA) in an ethanol solvent.

### 2.3. Synthesis of Silver Nanocrystals

**Figure 1:**
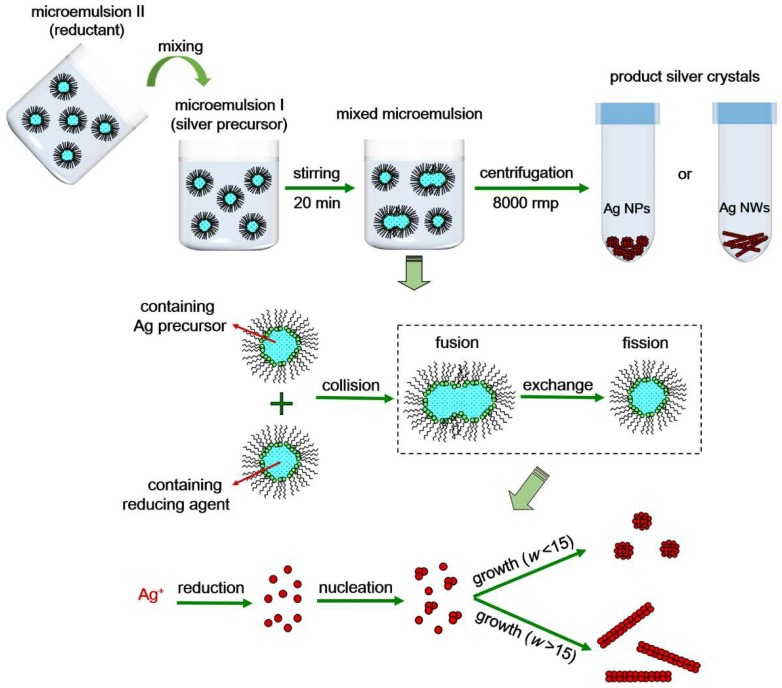
Schematic of the reaction process with a reverse microemulsion method.

Figure 1 describes the process of fabrication of our silver nanocrystals. For a typical synthesis (take the sphere-like silver nanoparticles (Ag NP) with a size of around 20 nm as an example), 0.70 g AOT was suspended in 50 mL of n-heptane in a conical flask to form a mixture duplicate with the mass fraction of AOT (W_AOT_) = 2%, which is realized by using a magnetic stirrer for 30 min. Next, 0.14 mL of silver nitrate solution (0.1 mol/L) was added into one of the two flasks, while 0.14 mL of ascorbic acid solution (0.3 mol/L) was added into the other flask. After 20 min stirring, two types of microemulsion solutions were formed. The microemulsion solution of ascorbic acid was then slowly added into the silver nitrate solution within 10 min. The entire solution was kept stirring for 3 h at ambient conditions to promote the reaction. The product was centrifuged at 8000 rpm for 10 min to gather the silver nanocrystals. After being washed with ethanol 3–5 times, the silver nanocrystals were cleaned (Figure S1) and stored in pure ethanol.

### 2.4. Characterization of Antibacterial Activity

The antibacterial activities of silver nanocrystals were investigated by MIC and MBC tests against *Escherichia coli* (*E. coli*) and *Staphylococcus aureus* (*S. aureus*). The fresh colonies were taken from an agar plate and inoculated into a 10 mL liquid Luria-Bertani (LB) broth media. Then, 0.1 μL of suspended bacteria (10^8^ CFU/mL) were inoculated into a 10 mL liquid LB media supplemented with 4096, 2048, 1024, 512, 256, 128, 64, 32, 16, 8, 4, 2, 1, 0.5, 0.25 and 0.125 μg/mL of silver nanocrystals. These samples with gradient concentrations were obtained by the double dilution method (see Supplementary Material for the detailed experimental process), and the silver nanocrystals were stable enough in liquid LB media for antibacterial measurements (Figure S2). A control experiment was performed using broth without silver nanocrystals. The samples were incubated at 37 °C and stirred at 200 rpm for 24 h. Growth rate was determined by measuring optical density (OD) at 600 nm at regular intervals. Evaluation of the MIC was done by visual inspection of growth/no-growth in mixtures containing different concentrations of the silver nanoparticles. Further, 100 μL of suspension were taken out from the tubes without visible bacteria growth and spread onto agar plates. The plates were then incubated at 37 °C for 24 h before the subsequent MBC measurements. The experimental details of MIC and MBC determination, as well as measurement of growth curves, are shown in Appendix A.

## 3. Results

**Figure 2:**
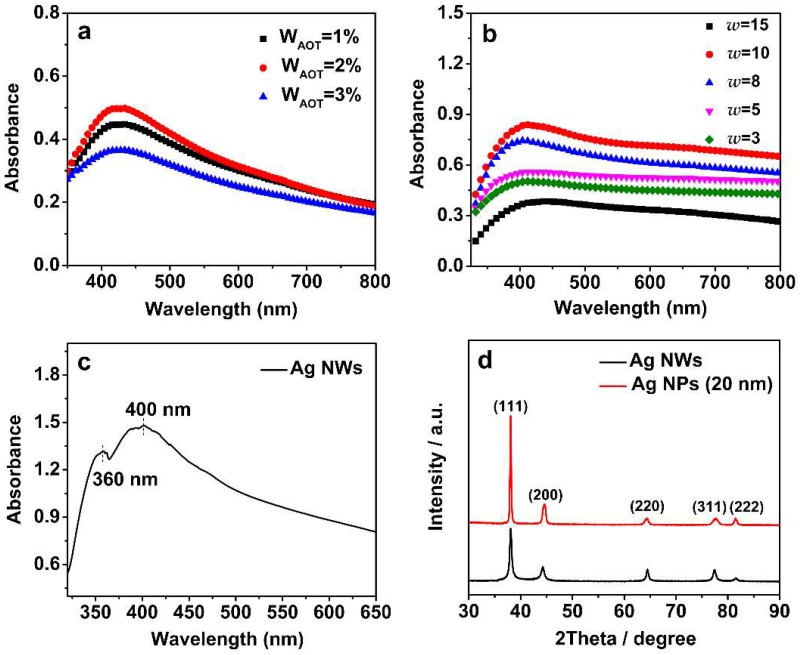
(**a**) Ultraviolet-visible spectroscopy (UV-vis) spectra of silver nanoparticles with different sodium bis(2-ethylhexyl) sulfosuccinate (AOT) mass fractions. UV-vis spectra of (**b**) spherical-like silver nanoparticles (Ag NP) synthesized at *w* = 15, 10, 8, 5, and 3 and (**c**) silver nanowires (Ag NW) synthesized at *w* = 25. (**d**) X-ray diffraction (XRD) of spherical-like Ag NP and Ag NW.

In the fabrication process, liquid media with nanosized water droplets were dispersed in the continuous oil phase (heptane) and stabilized by surfactant AOT molecules at water/oil interface. After mixing the two microemulsions, the water droplets collided with each other and there was an exchange of reactants. The liquid droplets then witnessed the nucleation reaction and growth of the silver nanocrystals. When the size of the as-prepared nanocrystal increased to be comparable with that of the liquid droplet, AOT molecules adsorbed onto the nanocrystals surface to prevent them from aggregating. Mass fraction of surfactant is a key parameter in the synthesis of nanocrystals by the reverse microemulsion method [67]. Surfactant is a necessary condition for nucleation during the synthesis. Nevertheless, excessive surfactants reduce the exchange of solute molecules between micelles, which leads to a lower yield. In order to find the optimized mass fractions of AOT (W_AOT_), we first carried out the UV-vis analysis of the nanocrystal products under different values of W_AOT_ and found that the critical value of W_AOT_ is between 1% and 3%. As shown in Figure 2a, the height of the peak increases as W_AOT_ increases from 1% to 2%, while the peak decreases in height as W_AOT_ increases further from 2% to 3%. Moreover, among the three curves, the peak in the 2% W_AOT_ curve has the smallest value of peak width at half-height (HWHM), indicating that the size distribution is more uniform than others. Thus, in subsequent experiments we adopted the 2% W_AOT_ as the condition for synthesis. 

In the microemulsion method, the size and morphology of the as-prepared nanocrystals can be controlled by the microreactors, i.e., the microemulsion droplets. Thus, we further investigated the influence of the molar ratio of water to AOT (*w*) on the synthesis of nanocrystals by UV-vis analysis in ethanol, as shown in Figure 2b,c. As the molar ratio increases from 3 to 15, the profiles of the adsorption peaks remain the same, while the peak height increases continuously. Notably, as the molar ratio increases further to 25, the profile of the UV-vis curve evolves to be a double peak, which indicates there is a transition in the morphology of the nanocrystal products.

**Figure 3:**
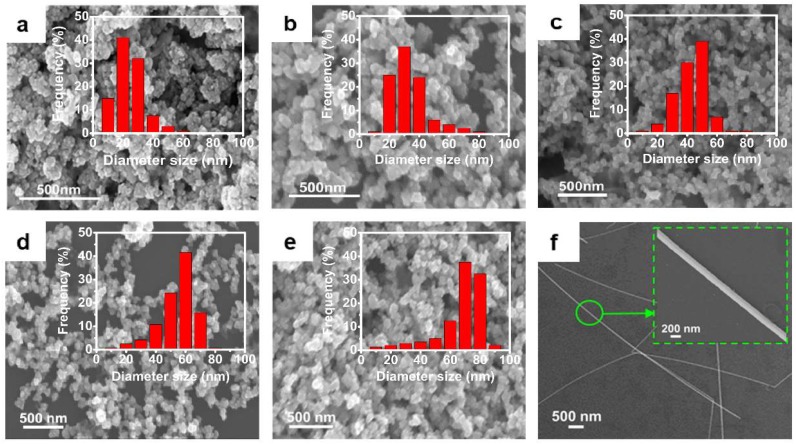
(**a**–**e**) Scanning electron microscopy (SEM) images of the Ag NP that synthesized at *w* = 3, 5, 8, 10 and 15, respectively. The inserts show the size distributions, which were measured by dynamic light scattering (DLS) in ethanol based on intensity and had been normalized to a frequency of size distribution. (**f**) SEM image of the Ag NW that synthesized at *w* = 25. In all six experiments, the mass fractions of AOT are 2%.

To study the transition of the morphology, the nanocrystals were determined by SEM, as shown in Figure 3. It is found that there is a transition from the sphere-like nanocrystal to the wire-like one as the molar ratio of water to AOT increases. With a small ratio, sphere-like silver nanoparticles (Ag NP) were formed, and the favorable diameter of Ag NP increases from 20 nm to 70 nm as the ratio (*w*) increases from 3 to 15. When the ratio (*w*) reaches 25, the assembly of these reagents forms silver nanowires (Ag NW) with a dimeter around 125 nm. The double peaks in Figure 2c can be ascribed to the Ag NW, which is in agreement with a previous report in Reference [68]. We then employed XRD analysis to identify the crystallographic structure and crystallinity of the silver nanocrystal products, as shown in Figure 2d. All diffraction peaks can be ascribed to face-centered cubic silver (JCPDS Card, No. 087-0597). Furthermore, there are no other peaks in the XRD spectra, indicating that our method is capable of preparing silver nanocrystals with high purity. Conventionally, there are two alternative products in the synthesis of nanocrystal: A spherical nanoparticle when a low surfactant concentration is adopted, or a non-spherical nanocrystal when a high surfactant concentration is adopted [69,70,71]. In this work, although we utilized a low surfactant concentration system, the size and the morphology of silver nanocrystals could be tuned conveniently by adjusting the ratio (*w*).

The antibacterial activities of the silver nanocrystal products against the *E. coli* and *S. aureus* were first evaluated through measurements of MIC and MBC, as shown in Table 1. As the starting molar ratio of water to AOT increases from 3 to 15, the silver nanocrystal products are sphere-like, i.e., Ag NP. It was found that the MIC and MBC values of the 20 nm Ag NP are lower than that of the 50 nm ones, indicating that the antibacterial activity of silver nanocrystal products decreases as the starting molar ratio increases. Nevertheless, as the molar ratio increases further to 25, the morphology transition of silver nanocrystal products occurs, and the MIC and MBC values against the two strains become lower than that of the 20 nm Ag NP.

**Figure 4:**
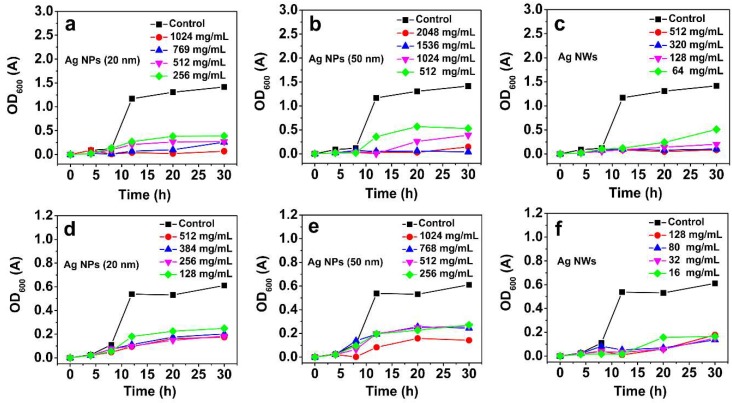
Growth curves of *E. coli* (**a**–**c**) and *S. aureus* (**d**–**f**) versus concentration.

To further investigate the antibacterial activities of the as-prepared nanocrystal products, we measured the growth curves of *E. coli* and *S. aureus* versus concentration, as shown in Figure 4. It was found that all of the silver nanocrystal products show high antibacterial activity, which was enhanced with the increase of the nanocrystal concentration. Additionally, the antimicrobial activity of silver nanoparticles was more effective against gram-positive bacteria *S. aureus* than that of the gram-negative bacteria *E. coli*. with the same concentration. The order of the as-prepared nanocrystal products by antibacterial activity is Ag NW, 20 nm Ag NP, and 50 nm Ag NP. Compared with the 50 nm Ag NP, the 20 nm size has a higher activity because it has a greater effective surface to load more silver ions [72]. Nevertheless, the Ag NW has the highest activity among the three nanocrystals, though it has the smallest effective surface, indicating that morphology plays a key role in the antibacterial activity of silver nanocrystals.

## 4. Conclusions

We developed a reverse microemulsion method to fabricate Ag nanocrystals by incorporation of H_2_O, n-heptane, and AOT. A variety of Ag nanocrystals with tunable sizes and morphologies were prepared and their antibacterial properties were investigated. As the molar ratio of water to surfactant is small, the resulted Ag nanocrystal products are sphere-like, and the antibacterial activity decreases as the size increases. As the molar ratio reached 25, the morphology of Ag nanocrystals evolved to be nanowire-like and exhibited a higher antibacterial activity than all of the sphere-like Ag nanocrystals. This work suggests that the morphology of silver nanocrystals plays a key role in the antibacterial process. Our method provides a simple, economic, and effective approach to preparing nanoparticles, which has promising applications in both material science and food industry.

## Figures and Tables

**Table 1:** The minimum inhibitory concentration (MIC) and minimum bactericidal concentration (MBC) values of the as-prepared nanocrystal products.

Bacteria	MIC (μg/mL)			MBC (μg/mL)		
	Ag NP (20 nm)	Ag NP (50 nm)	Ag NW	Ag NP (20 nm)	Ag NP (50 nm)	Ag NW
*E. coli*	512	1024	128	1024	2048	512
*S. aureus*	256	512	32	512	1024	128

## Supplementary Materials

The following are available online at http://www.mdpi.com/1996-1944/11/12/2498/s1, Figure S1: Thermogravimetric analysis (TGA) of (**a**) pure surfactant AOT and (**b**) silver nanoparticle synthesized with the molar ratio of water to AOT *w* = 3, Figure S2: UV-vis spectra of as-prepared silver nanoparticles synthesized with the molar ratio of water to AOT *w* = 3 within 24 h.

## Appendix A

### Appendix A.1. MIC (Minimum Inhibitory Concentration)

The antibacterial activity of a biomaterial is described in terms of MIC. In this work, MIC refers to the lowest concentration of the silver nanocrystal capable of inhibiting bacterium growth. Before the MIC measurement, the glassware and culture medium were sterilized (0.1 MPa steam, 121 °C, for 20 min). A series of silver nanocrystal solutions with gradient concentrations were obtained by the double dilution method. Then 0.1 μL of 10^8^ colony-forming units (CFU)/mL bacteria suspension was added into each of the silver nanocrystal solutions. All the operations were conducted under sterile circumstances. Finally, the inoculated microdilution tubes were covered with silicone stoppers and incubated at 37 °C for 24 h under shaking conditions. Evaluation of the MIC was then done by visual inspection of growth/no-growth in the mixtures containing different amounts of silver nanoparticle.

### Appendix A.2. MBC (Minimum Bactericidal Concentration)

MBC is another parameter to describe the antibacterial activity of a biomaterial. In this work, it refers to the lowest concentration of the silver nanocrystal capable of killing the organism. After the MIC measurements, we continued to determine the MBC of our silver nanocrystals. To evaluate the MBC, a 100 μL solution without visible bacterium was removed from the tubes and coated onto agar plates. The plates were then incubated at 37 °C for 24 h. Then, evaluation of the MBC was done by a visual inspection when there is no surviving strain observed on the agar plates.

### Appendix A.3. Growth Curve

Growth curve describes the growth of microorganisms, i.e., *Escherichia coli* and *Staphylococcus aureus* herein, with antibacterial agents. In this work, for each of the probe solutions, the growth curves were obtained by monitoring the evolution of optical density (OD) over 30 h, which is performed by an ultraviolet-visible spectroscope with an incident light of 600 nm wavelength.
