# Supplementary material for: Facile Synthesis of Silver Nanoparticles with High Antibacterial Activity
Source: Materials (Basel). 2018 Dec 8;11(12):2498. doi: 10.3390/ma11122498 (PMC6316916; doi:10.3390/ma11122498)
Supplement: Supplementary file 1 [file materials-11-02498-s001.pdf]

materials

Supplementary Materials: Facile Synthesis of Silver Nanoparticles with High Antibacterial Activity

Anni Feng, Jiankang Cao, Junying Wei, Feng Chang, Yang Yang and Zongyuan Xiao

1. Preparation Method of Silver Solutions with Different Concentrations in Antibacterial Experiment

In our experiment, a series of solution with a concentration gradient were obtained by the double dilution method. First, we prepared 18 clean test tubes that were labelled as No. 1 to No. 18. In each of the tubes, we added 10 mL Luria-Bertani (LB) broth media. Second, 81.92 mg silver sample was suspended in the No. 1 test tube to form a stock solution, in which the concentration of silver nanocrystal is as high as 8192 μg/mL. Third, 10 mL of the No.1 tube solution was transferred to the No. 2 tube. Thus, in the No. 2 tube, we obtained a 20 mL solution with a concentration of 4096 μg/mL. Then, 10 mL of the No. 2 tube solution was transferred to No. 3 tube. And so forth, we obtained the other 15 solutions with decreasing concentrations. Specifically, their concentrations of silver nanocrystal are 2048 μg/mL (No. 3), 1024 μg/mL (No. 4), 512 μg/mL (No. 5), 256 μg/mL (No. 6), 128 μg/mL (No. 7), 64 μg/mL (No. 8), 32 μg/mL (No. 9), 16 μg/mL (No. 10), 8 μg/mL (No. 11), 4 μg/mL (No. 12), 2 μg/mL (No. 13), 1 μg/mL (No. 14), 0.5 μg/mL (No. 15), 0.25 (No. 16) and 0.125 μg/mL (No. 17), respectively. In the No. 18 tube, there is just 10 mL LB broth medium. Thus, its concentration of silver nanocrystal is 0. In our experiment, the No. 18 tube solution was used as the control solution.

2. .Thermogravimetric Analysis (TGA) for Exclusion of the Organic Contaminants on the As-Prepared Ag NPs Surface

To exclude the presence of organic contaminants on the silver nanoparticles surface we carried out the TGA measurement for pure surfactant AOT, as shown in Figure S1a. Then, we carried out the TGA measurement for a typical silver nanocrystal sample, for which the molar ratio of water to AOT is 3, i.e., $w = 3$, as shown in Figure S1b. Compared with Figure S1a, in Figure S1b there is no feature of organic contaminants in the thermogravimetric (TG) curve or the differential thermalgravity (DTG) curve. Based on this supplementary experiment, we can exclude the presence of organic contaminants on the NPs surface.

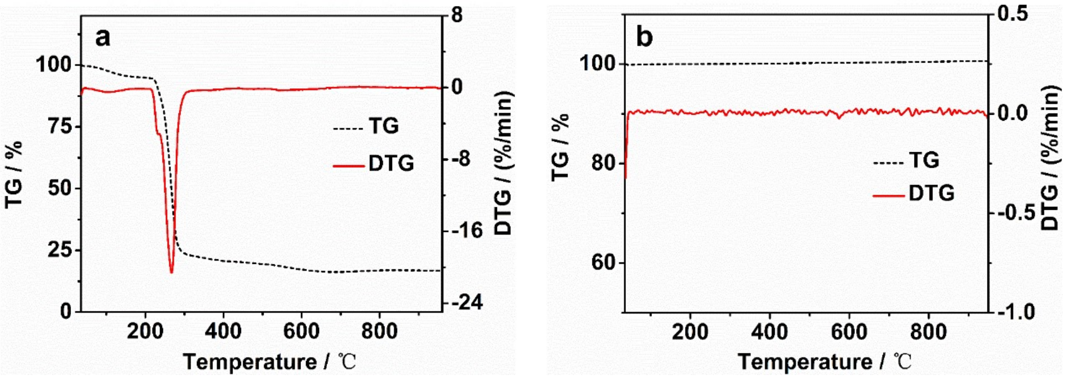

Figure S1. Thermogravimetric analysis (TGA) of (**a**) pure surfactant AOT and (**b**) silver nanoparticle synthesized with the molar ratio of water to AOT $w = 3$.

3. Stability Measurement of As-Prepared Silver Particles

The as-prepared silver nanocrystal samples were characterized by UV-vis measurement in LB broth media. It is found that after 24 h, both the profile and peak shift of the absorbance is almost

unchanged. Thus, the as-prepared silver nanocrystals are stable enough for the antibacterial experiments.

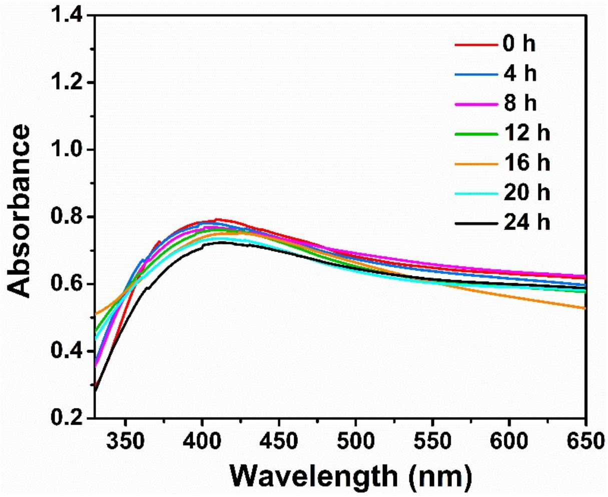

Figure S2. UV-vis spectra of as-prepared silver nanoparticles synthesized with the molar ratio of water to AOT w = 3 within 24 h.
